# Two-channel Hyperspectral LiDAR with a Supercontinuum Laser Source

**DOI:** 10.3390/s100707057

**Published:** 2010-07-23

**Authors:** Yuwei Chen, Esa Räikkönen, Sanna Kaasalainen, Juha Suomalainen, Teemu Hakala, Juha Hyyppä, Ruizhi Chen

**Affiliations:** 1 Department of Navigation and Positioning, Finnish Geodetic Institute, P.O. Box 15 02431 Masala, Finland; E-Mail: ruizhi.chen@fgi.fi; 2 Department of Remote Sensing and Photogrammetry, Finnish Geodetic Institute, P.O. Box 15 02431 Masala, Finland; E-Mails: esa.raikkonen@iki.fi (E.R.); Sanna.Kaasalainen@fgi.fi (S.K.); juha.suomalainen@fgi.fi (J.S.); teemu.hakala@fgi.fi (T.H.); juha.hyyppa@fgi.fi (J.H.); 3 Klastech GmbH, Konrad-Adenauer-Allee 11,D-44263 Dortmund, Germany; E-Mail: E.Raeikkoenen@klastech.de

**Keywords:** hyperspectral LiDAR, prototype, range finder, NDVI

## Abstract

Recent advances in nonlinear fiber optics and compact pulsed lasers have resulted in creation of broadband directional light sources. These supercontinuum laser sources produce directional broadband light using cascaded nonlinear optical interactions in an optical fibre framework. This system is used to simultaneously measure distance and reflectance to demonstrate a technique capable of distinguishing between a vegetation target and inorganic material using the Normalized Difference Vegetation Index (NDVI) parameters, while the range can be obtained from the waveform of the echoes. A two-channel, spectral range-finding system based on a supercontinuum laser source was used to determine its potential application of distinguishing the NDVI for Norway spruce, a coniferous tree, and its three-dimensional parameters at 600 nm and 800 nm. A prototype system was built using commercial components.

## 1. Introduction

Light Detection And Ranging (LiDAR) instruments make use of the extreme directionality of laser light, which enables measurement of distances with high spatial accuracy. Airborne Laser Scanning (ALS) is based on LiDAR range measurements conducted between an aircraft and the object of study [1]. Recent ALS instruments mostly use monochromatic lasers to measure surface topography and for object characterization. ALS produces a 3D point cloud (x,y,z) of the surveyed target area, and the cloud represents the coordinates of the object. The intensity (I) value is also recorded for each point, either as the echo amplitude (proportional to the number of photons received by the detector over a given period of time) or as the complete echo waveform [2,3]. Traditionally, the intensity data has mainly been used for matching images and laser strips, and rough classification and recognition of points and objects. However, recent the improvements in radiometric calibration [2–6] have significantly improved the use of the data. The use of Terrestrial Laser Scanners (TLS) has also increased, and the number of applications, as well as information on TLS performance and range-data accuracy, is constantly increasing [7].

Recent advances in nonlinear fibre optics and compact pulsed lasers have brought to the market light sources that are extremely broadband, yet as directional as laser light. These supercontinuum laser sources produce directional broadband light by making use of cascaded nonlinear optical interactions in an optical fibre [8,9], and they can be used to simultaneously measure distance and the reflectance spectrum, which has been the basis for recent efforts at developing hyperspectral LiDAR [10].

Current techniques for producing 3D point clouds with spectral intensity information are based on combining the laser-scanner data with passive spectroscopic sensing, e.g., aerial images or passive imaging spectrometry [11], or using separated semiconductor laser diodes as the laser source [12]. These approaches enable the classification of laser points for object recognition, but there are several practical problems. These include discrete spectral band, inaccuracy in registration, and time-based variation between measurements. Also, active hyperspectral imaging applications without range information have been developed [10]. The idea behind hyperspectral LiDAR is that it would enable simultaneous hyperspectral imaging and laser scanning by the same instrument without any registration problems arising between the data sets, and it would produce a point cloud combined with hyperspectral intensity [x,y,z,I(λ)], where I(λ) represents intensity (I) as a continuous function of wavelength (λ).

The objective of this paper is to demonstrate the use of a spectral range-finding system using two channels in two different applications: determination of the Normalized Difference Vegetation Index (NDVI) of a Norway Spruce (*Picea abies*) tree, and obtaining the target range. The NDVI is defined as: NDVI = [NIR-RED]/[NIR+ RED], where NIR is the target reflectance within the near-infrared wavelength range, and RED is the reflectance within the visible wavelength range. The system is different from the currently available dual or multi wavelength LiDAR applications because the wavelengths can be selected from within the supercontinuum (or detector) wavelength range. The system configuration is presented in Section 2. The experiments are outlined in Section 3, and the Results and Discussion are provided in Section 4.

## 2. System Configuration

**Figure 1.**
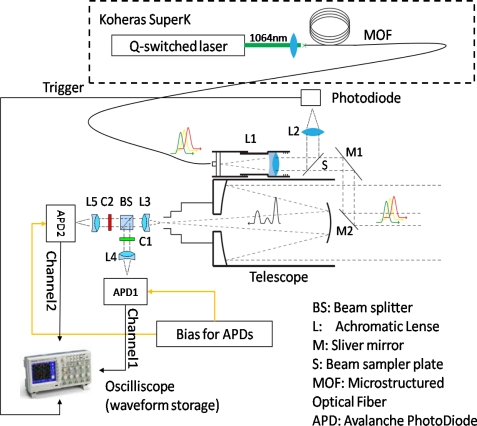
Schematic setup of two-channel hyperspectral LiDAR.

Figure 1 presents the schematic setup of the two-channel hyperspectral LiDAR. The light source used is a supercontinuum laser source (Koheras SuperK) with a wavelength range of 600 to about 2,000 nm, and an average output power of 100 mW [13]. The pulse rate is 20–40 kHz with 1–2-ns pulse width. The “white” laser pulse is emitted from a Microstructured Optical Fibre (MOF) with a high divergence angle. Therefore, it is collimated using an achromatic lens (L1 in Figure 1) before being transmitted to the object. The collimated beam is reflected towards the target by means of two silver mirrors (M1 and M2 in Figure 1). Mirror M2 is placed at the optical axis of the receiving telescope. A photodiode sensor is situated beside a beam sampler plate (S in Figure 1), which is used to collect the sample of the transmitting laser beam reflected from the beam sampler plate. The collected signal is applied to trigger a high-speed oscilloscope to archive the waveforms of both the transmitting pulse and the receiving echoes, whereby the time-of-flight measurement could be carried out using post-processing software.

A Cassegrain telescope (with 1,000 mm focal length, 10 cm aperture diameter) collected the scattered laser pulse from the target. The focal point of the telescope is imaged onto two avalanche photodiode sensors (APD1 and APD2 in Figure 1) via a beam splitter (BS in Figure 1) and three achromatic lenses (L3 L4 and L5 in Figure 1). The split beam is filtered using a band-pass colour filter (C1 and C2 in Figure 1 with a bandwidth of 40 nm and a peak transmission of 600 nm and 800 nm for channel1 and channel2, respectively). The filtered beam is focused via the achromatic lens onto the high-voltage-biased APD sensor, which converted the laser echo into an electronic signal. The output signals from the APD sensors were sampled and recorded using the high-speed oscilloscope (5 gigapoints per second sampling rate, 500 MHz band width, four channels), resulting in a set of spectrally-resolved waveform echoes (two channels in this case). Therefore, the prototype system measured the time of flight and the spectrum of the returning echoes by post-processing the recorded waveform from each channel.

Purpose of this experiment is to demonstrate the instrument’s ability to distinguish vegetation (the Norway spruce target) from inorganic material by means of the NDVI parameter. The spectral response of the APD sensor is typical for a silicon avalanche photodiode with peak spectral response at 800 nm. There is typically an increase in reflectance at about 700 nm for vegetation targets, resulting in NDVI values greater than zero [11,13]. Furthermore, the characteristic of the peak spectral response at 800 nm can increase the S/N (Signal-to-Noise) ratio of the near-infrared measurement. Therefore, the spectral bands of 600 nm and 800 nm provide the best S/N ratio and distinguishing Norway spruce from organic matter.

Using the beam splitter and band-pass optical filter configuration, it is possible to modify the system for several specific experiments simply by changing the band-pass optical filter. The current configuration can be easily extended to encompass a four-channel solution only if the same receiving optical configuration is duplicated and the response echoes are filtered by a band-pass filter.

## 3. Methods

**Figure 2.**
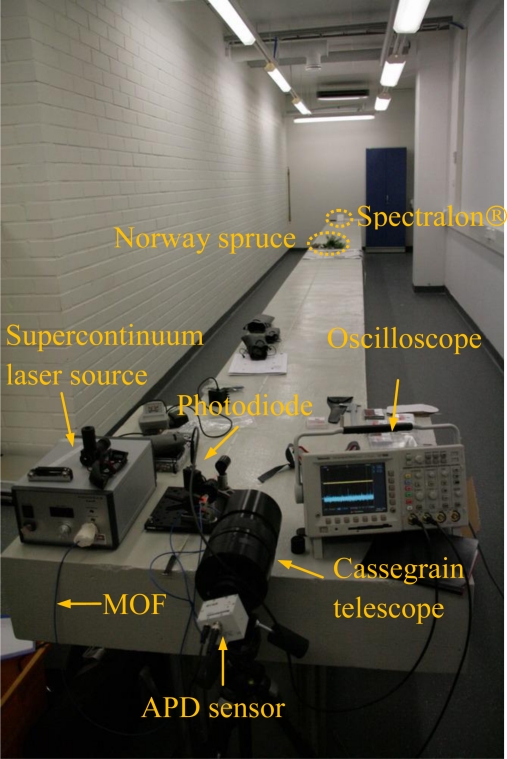
Hyperspectral LiDAR demonstration instrument used in the laboratory test.

The two-channel hyperspectral LiDAR was field tested at the Geodesy Laboratory of the Finnish Geodetic Institute in December 2009 (see Figure 2). The laboratory has a 12-metre concrete table, which enabled the targets to be placed at a measured distance (∼11 metres) from the laser source. Two sets of experiments are designed to measure the precision accuracy of the hyperspectral LiDAR system. The goal of these experiments is to demonstrate the feasibility and applications of hyperspectral range-finding measurements and to evaluate the performance of the prototype system.

The first experiment evaluated the performance of the system as a laser range finder based on time-of-flight measurements. A Spectralon® (Labsphere Inc.) reflection board (reflection rate: 99%) is placed at a position of 11 metres from the aperture of the telescope along the optical axis of the telescope. The target is replaced at a position of 10 metres and the time difference between these two measurements is determined using a high-speed oscilloscope.

The feasibility of simultaneous range and spectral measurements is demonstrated in the second experiment. A Norway spruce tree target is placed at a distance of about 9.3 metres in front of the Spectralon® reflection board, which is placed at a distance of 10.7 metres. The transmitting laser pulse passed through the branches of the spruce tree and is projected onto the reflection board. The main part of the pulse is reflected by the spruce and the remaining part by the Spectralon® panel, thus producing multiple echoes. This enabled the distances and reflectances at 600 nm and 800 nm for both targets to be compared.

## 4. Results and Discussion

**Figure 3.**
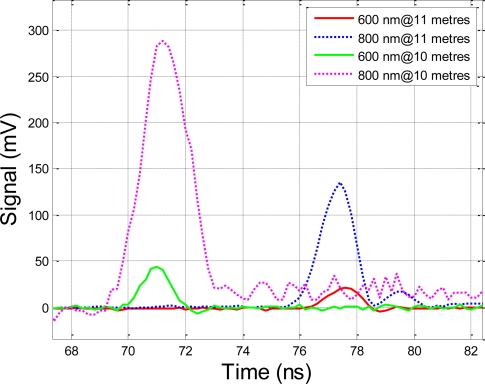
The echoes of two-channel hyperspectral LiDAR from the Spectralon® reflection board at ranges of 10 and 11 meters, respectively

The results of the time-of-flight experiment are illustrated in Figure 3, which shows the echoes from the two APD sensors at both target distances. Assuming the peak position of the echo waveform represents the range of the target, both channels indicate a range of 11.625 metres and 10.665 metres (which correspond to 77.5 ns and 71.1 ns, respectively, as the measured time-of-flight values). The echo of the 800 nm band is higher than that of the 600 nm band, which is caused by the combined effect of the stronger laser output and the higher spectral response of the APD sensor on the 800 nm band.

The measured range difference between the two measurements is slightly less than 1 metre, while the actual difference is 1 metre during the experiments. The following are explanations for what may have caused the inaccuracy in the distance measurement:

1. The sampling rate of the oscilloscope is 5 gigapoints per second, *i.e.,* the range resolution of the system in this case is 3 cm (0.2 ns), resulting in a 3 cm random quantization error for every single range measurement and at maximum an error of 6 cm for range difference measurement.
2. The bandwidth of the oscilloscope is 500 MHz, which is slightly less than that of the echoes, and this may partly distort the echo waveform.
3. The oscilloscope is triggered by the raising edge of the transmitting pulse signal collected by the photodiode at a fixed trigger level. The power of the transmitting pulse varied during the experiment. The increase in the echo amplitudes is not proportional to the distance, as can be observed in Figure 3 by comparing the echoes at different distances. The variance of transmitting power caused the shift in the raising edge of the triggered signal. As a result, an extra error is introduced into distance measurements.
4. The results are based on the stored waveform of the high-speed oscilloscope, but not on a high-precision, time-interval measurement instrument. However, an oscilloscope is not designed for time-of-flight measurements and the oscilloscope applied in these tests has never been calibrated for such a purpose. It is used here only to demonstrate the time-of-flight measurement. A high-time-resolution frequency counter is preferable for an application such as this.
5. The concrete table on which the prototype system set is not meant for precise optical measurements. Consequently, the reference measurement of distance is carried out on a rough surface, and this may introduce some degree of error.

**Figure 4.**
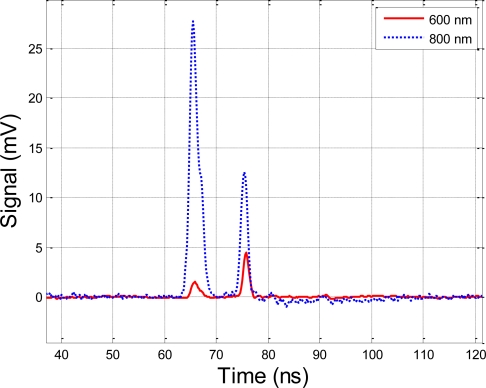
The echoes of the two-channel hyperspectral LiDAR for two targets (Spectralon® panel behind the Norway spruce tree), averaged for 16 measurements.

**Figure 5.**
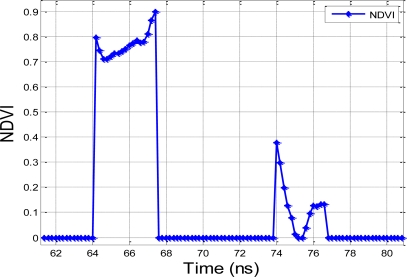
The NDVI of the echoes of the two-channel hyperspectral LiDAR for two targets (Spectralon® panel behind the Norway spruce tree, cf. Figure 4), averaged for 16 measurements and the echoes after normalization.

Figure 4 shows the multiple echoes from the target spruce tree and the Spectralon® panel in the second experiment. Since the transmitting power varied during the test, we calculated the results by averaging 16 measurements to mitigate the effect of this variation and to improve the S/N ratio. We observed the following phenomena:

1. The prototyping system is capable of processing multiple hits, which it can extract from multiple targets from one echo. This is a critical feature for forestry applications.
2. The difference in the NDVI for the Spectralon® reflection board and the Norway spruce tree sample is clearly visible in this experiment (see Figure 5). The NDVI of the Norway spruce tree is about 0.8 and the curve is moderately flat. Likewise, the NDVI of the Spectralon® panel is fairly close to 0, which is in agreement with the Spectralon panel having a flat reflection rate at all wavelengths. The authors approximated the near-infrared and visible parts of the spectrum with the reflectance values at 800 nm and 600 nm, respectively. The spikes in the NDVI curve for the Spectralon® panel at 74 and 77 ns are caused by the uneven spectral response feature between two APD sensors, which can be calibrated when conducting future research. The results are in agreement with earlier observations of high NDVI values for vegetation [11,13].

Nevertheless, these results demonstrate the feasibility of the hyperspectral range finder for simultaneous range and spectral measurements: the instrument is capable of discriminating between a Norway spruce target and inorganic material based on the NDVI spectral index, while at the same time obtaining the target’s range. The statistical parameters of the echo signals are analyzed and listed in Table 1. The following are some facts to be concluded from Table 1.

1. The S/N (Signal-to-Noise) ratio for all tests is promising.
2. The S/N ratio is higher at 800 nm than at 600 nm. This is caused by the combined effects of the stronger laser output and the higher spectral response of the APD sensor at 800 nm.
3. The S/N ratio increases by averaging multiple echo measurements.

The proposed system can be easily expanded into a four-channel hyperspectral LiDAR, thereby enabling more complex scientific experiments.

Future improvements and calibration designs will also improve the accuracy of the results. The oscilloscope is more efficient at monitoring the signal than storing the waveform. An oscilloscope with wider bandwidth (1–2 GHz bandwidth) is needed to mitigate the distortion for the echo signal. A high-time-resolution frequency counter will be introduced into the system to provide precise time-of-flight measurements. Although a band-pass optical filter may be appropriate for laboratory tests, other solutions should also be explored; e.g., a grating-based system may be a more practical method, especially for an outdoor experiment. Since most commercial high-speed waveform digitalizing products offer 8-bit A/D (Analog-to-Digital) converting capability, the dynamic range of such products should be considered. Assuming the target distance varies from 1–20 metres, an 11-bit product is needed for an appropriate solution. Furthermore, a functional calibration solution is essential due to the following observations:

1. Spectrum deviation of the transmitting pulse occurs during the operation.
2. The transmitting power of the laser pulse varies.
3. The spectral response feature for each APD sensor is not identical.

## 5. Conclusions

The two-channel hyperspectral LiDAR prototype successfully carried out a series of experiments to demonstrate the feasibility of the concept in both range-finding and spectral reflectance measurement. To the authors’ knowledge, this is one of the first applications of the newly commercialized supercontinuum laser technology. This is also the first demonstration of the concept of an active hyperspectral LiDAR, which provides simultaneous range and spectral information. Thus far, the combination of 3D and hyperspectral remote sensing data has been based on passive spectral measurements. Determination of the NDVI of a Norway spruce tree was demonstrated and the results agree well with those from the literature on passive imaging based on backscatter. Future work aims at expanding the spectral range of the detector part by increasing the number of channels, as well as developing a fully functional hyperspectral LiDAR instrument.

## Figures and Tables

**Table 1.** The statistical parameters of the recording echo waveform. RMS = root mean square error.

**Test**	**Channel 1 (600nm)**			**Channel 2 (800nm)**		
	**RMS of noise [mV]**	**Maximum signal [mV]**	**S/N ratio**	**RMS of noise [mV]**	**Maximum signal [mV]**	**S/N ratio**
Spectralon at 11 m	0.567	21.6	38.1	0.764	134.9	176.6
Spectralon at 10 m	1.7	44.1	25.9	7.4	288.3	39.0
NDVI test (average of 16 measurements)	0.066	4.05	61.4	0.13	27.8	213.8
